# The Glasgow Prognostic Score, an inflammation based prognostic score, predicts survival in patients with hepatocellular carcinoma

**DOI:** 10.1186/1471-2407-13-52

**Published:** 2013-02-02

**Authors:** Akiyoshi Kinoshita, Hiroshi Onoda, Nami Imai, Akira Iwaku, Mutumi Oishi, Ken Tanaka, Nao Fushiya, Kazuhiko Koike, Hirokazu Nishino, Masato Matsushima, Chisato Saeki, Hisao Tajiri

**Affiliations:** 1 Division of Gastroenterology and Hepatology, The Jikei University Daisan Hospital, 4-11-1 Izumihon-cho, Komae- shi, Tokyo, 201-8601, Japan; 2 Division of Clinical Epidemiology, The Jikei University School of Medicine, 3-25-8 Nishishinbashi, Minato-ku, Tokyo, 105-8461, Japan; 3 Division of Gastroenterology and Hepatology, Department of Internal Medicine, The Jikei University School of Medicine, 3-25-8 Nishishinbashi, Minato-ku, Tokyo, 105-8461, Japan

**Keywords:** The Glasgow Prognostic Score, Hepatocellular carcinoma, Prognostic marker

## Abstract

**Background:**

Elevated Glasgow Prognostic Score (GPS) has been related to poor prognosis in patients with hepatocellular carcinoma (HCC) undergoing surgical resection or receiving sorafenib. The aim of this study was to investigate the prognostic value of GPS in patients with various stages of the disease and with different liver functional status.

**Methods:**

One hundred and fifty patients with newly diagnosed HCC were prospectively evaluated. Patients were divided according to their GPS scores. Univariate and multivariate analyses were performed to identify clinicopathological variables associated with overall survival; the identified variables were then compared with those of other validated staging systems.

**Results:**

Elevated GPS were associated with increased asparate aminotransferase (P<0.0001), total bilirubin (P<0.0001), decreased albumin (P<0.0001), α-fetoprotein (P=0.008), larger tumor diameter (P=0.003), tumor number (P=0.041), vascular invasion (P=0.0002), extra hepatic metastasis (P=0.02), higher Child-Pugh scores (P<0.0001), and higher Cancer Liver Italian Program scores (P<0.0001). On multivariate analysis, the elevated GPS was independently associated with worse overall survival.

**Conclusions:**

Our results demonstrate that the GPS can serve as an independent marker of poor prognosis in patients with HCC in various stages of disease and different liver functional status.

## Background

Hepatocellular carcinoma (HCC) is the seventh most common cancer worldwide, and the third leading cause of cancer-related deaths [1]. In contrast to other cancers, prognosis and treatment options for patients with HCC depend not only on the tumor progression but also on the extent of liver dysfunction [2].

A number of staging systems for HCC have been proposed including Barcelona Clinic Liver Cancer (BCLC) [3], Cancer Liver Italian Program (CLIP) [4], and Japanese Integrated Staging Score (JIS) systems [5]. However, a worldwide consensus has not been established on which of the systems is most accurate for staging and predicting prognosis of HCC.

In addition, accumulating evidence indicates that the Glasgow Prognostic Score (GPS) system based on inflammation criteria and including only serum C-reactive protein (CRP) and albumin, is a reliant and practical scoring system for outcome prognostication in patients with advanced cancer, such as colorectal cancer [6,7], esophageal cancer [8], gastric cancer [9], pancreatic cancer [10], and lung cancer [11]. Recently, Proctor et al. have shown that modified GPS (mGPS) is a powerful prognostic factor independent of tumor site in patients with cancer and is superior to GPS [12]. It was based on the observation that hypoalbuminaemia without an elevated CRP concentration was rare and that hypoalbuminaemia on its own was not associated with poor survival [13].

In regard to patients with HCC, Ishizuka et al. have demonstrated that GPS can serve as a predictor of overall survival but the patients enrolled in their study included only those who underwent surgical resection [14]. Morimoto et al. also have shown that elevated GPS has a significant prognostic value in patients with advanced HCC, but the study was limited to patients treated with sorafenib [15]. Thus, although the studies addressed validity of GPS in HCC patients, they did not provide sufficient evidence whether elevated GPS is prognostically efficient in all HCC patients, *i*.*e*. with different stages of the disease and different liver functional statuses, and did not clarify which of the GPS (original or modified) is more suitable in regard to their discriminating ability and monotonicity of gradients.

In the present study, we evaluated usefulness of both GPS and mGPS in prediction of overall survival in patients with HCC in various stages of the disease and different liver functional statuses, and compared obtained findings with those of other validated staging systems.

## Methods

### Patients

Two hundred and eight consecutive patients with newly diagnosed HCC treated at the Department of Gastroenterology and Hepatology, Jikei University Daisan Hospital, between January 2005 and October 2011 were prospectively enrolled and their medical records were retrospectively reviewed. Twenty-three patients were lost to follow up. Thirty-five patients, whose entire set of laboratory data was not available, were excluded from the study. Patients who showed clinical evidence of infection or other inflammatory conditions were also excluded. In total, 150 patients with HCC were finally enrolled and evaluated; all were included in our previous study [16].

The diagnosis of HCC was pathologically confirmed or was based on findings obtained by 4-phase multidetector computed tomography (CT) or dynamic contrast-enhanced magnetic resonance imaging (MRI). Definitive diagnosis was made when a typical hallmark of HCC (hypervascular area in the arterial phase and washout area in the portal venous or delayed phases) [17] was observed in the contrast-enhanced images. Tumor-related variables such as the maximal tumor diameter, number, vascular invasion, and extra hepatic metastases were evaluated with the same imaging techniques. The clinical stage (TNM classification) was determined according to the Liver Cancer Study Group of Japan [18].

This study complied with the standards of the Helsinki Declaration and current ethical guidelines and was approved by the institutional ethical board of the Jikei University Daisan Hospital. Written informed consent for participation in the study was not obtained from patients, because this study did not report on a clinical trial, and the data ware retrospective in nature and analyzed anonymously.

### GPS and other variables

Blood samples were obtained before commencement of treatment for CRP, serum albumin, asparate aminotransferase (AST), alanine aminotransferase (ALT), total bilirubin (T-Bil), white blood cell count (WBC), platelet count (Plt), prothrombin time (PT), indocyanine green dye retention rate at 15 minutes (ICG), and α-fetoprotein levels (AFP). The CLIP score, JIS score, BCLC were calculated based on obtained results and imaging data.

GPS and mGPS were described previously. Briefly, in GPS, patients with both an elevated CRP level (>1.0 mg/dl) and hypoalbuminemia (<3.5 g/dl) were allocated a score of 2, patients with only one of these biochemical abnormalities were allocated a score of 1, and patients with neither of these abnormalities were allocated a score of 0 [19]. The mGPS was calculated also with CRP and albumin values as follows: patients with both an elevated CRP level (>1.0 mg/dl) and hypoalbuminemia (<3.5 g/dl) were allocated a score of 2, patients with an elevated CRP level (>1.0 mg/dl) only were allocated a score of 1, and patients with a normal CRP level (≤ 1.0 mg/dl) and any albumin concentration were allocated a score of 0 [20].

### Treatment and patient’s follow-up

Criteria of surgical resection were: a solitary lesion, Child-Pugh grade A, no main portal vein trunk involvement or no distant metastasis. Radiofrequency ablation (RFA) or percutaneous ethanol injection (PEI) was performed in patients with lesions <3 cm in size and <3 in number. Transcatehter arterial chemoembolization (TACE) or lipiodol- transcatehter arterial infusion (TAI) was performed in patients with multiple lesions of more than 4 in number or larger than 3 cm in size. Systemic chemotherapy or targeted therapy including sorafenib was carried out in patients with distant metastasis but preserved liver function. For patients with Child-Pugh grade C or distant metastasis, only best supportive care (BSC) was provided. We presumed that it is of importance to evaluate prognosis in patients with curative or non-curative treatment separately in an attempt to minimize the impact of different treatment modalities in the process of evaluating the prognostic model. Therefore, in this study, according to the current EASL-EORTC clinical practice guidelines [17], a curative treatment was defined as aggressive treatment, including surgical resection, RFA, PEI. By contrast, a non-curative treatment was defined as other palliative treatment (TACE, TAI, systemic chemotherapy, sorafenib or BSC).

After the initial treatment phase, patients were carefully followed. Serum AFP was measured once every month. Ultrasonography and dynamic CT were performed every 3 months. A selective hepatic arterial angiography or a percutaneous biopsy was performed in patients with suspected tumor recurrence. The start date of follow-up period was the date of initial HCC diagnosis. The end of the follow-up was set as the time of last follow-up (October 2011) or death.

### Statistical analysis

Continuous variables were presented as median and range. Categorical variables were presented numbers and percentages. Comparison between the groups was performed with the Kruskal-Wallis test for continuous and ordinal variables and with the chi-square test for categorical variables. The overall survival rates were calculated using the Kaplan-Meier method and differences in the survival rates between the groups were compared by the log-rank test. To compare the prognostic ability of each staging systems, the linear χ^2^ test (for measuring both discriminatory ability and monotonicity of the gradient across categories) and the −2 log likelihood (for measuring homogeneity) were used [21,22]. Both tests were performed using values calculated from the Cox-proportional hazard model. Generally, more accurate staging systems showed higher linear χ^2^ values and lower −2 log likelihoods. To evaluate the discriminatory ability of each staging systems, a receiver operating characteristics (ROC) curves was generated and the areas under the curve were measured. For assessment of prognostic factors, univariate and multivariate analysis were performed using the Cox-proportional hazard model. Variables that proved to be significant in the univariate analysis were tested subsequently with multivariate Cox-proportional hazard model. The forward selection method was used for multivariate Cox-proportional analysis. A P-value <0.05 was considered significant. All statistical analyses were performed using IBM SPSS Statistics software v.19.0 (IBM SPSS Inc., Chicago, IL, USA).

## Results

### Patient characteristics

The median age of patients was 72 (range 43–91) years. One hundred and six (70.7%) patients were males and 44 (29.3%) patients were females. Eighty four (56%) patients were positive for antibodies to hepatitis C virus (anti-HCV), 20 (13.3%) patients were positive for hepatitis B surface antigen (HBs Ag). Diagnosis of HCC was confirmed histologically in 31 (20.7%) patients, whereas in other patients it was based on imaging data. Surgical resection was performed in 9 (6%) patients, TACE or RFA were carried out in 134 (89.3%) patients. One (0.7%) patient received systemic therapy. The remaining 6 (4%) patients received BSC. Seventy seven (51.3%) patients received curative treatment and 73 (48.7%) patients received non-curative treatment.

**Figure 1:**
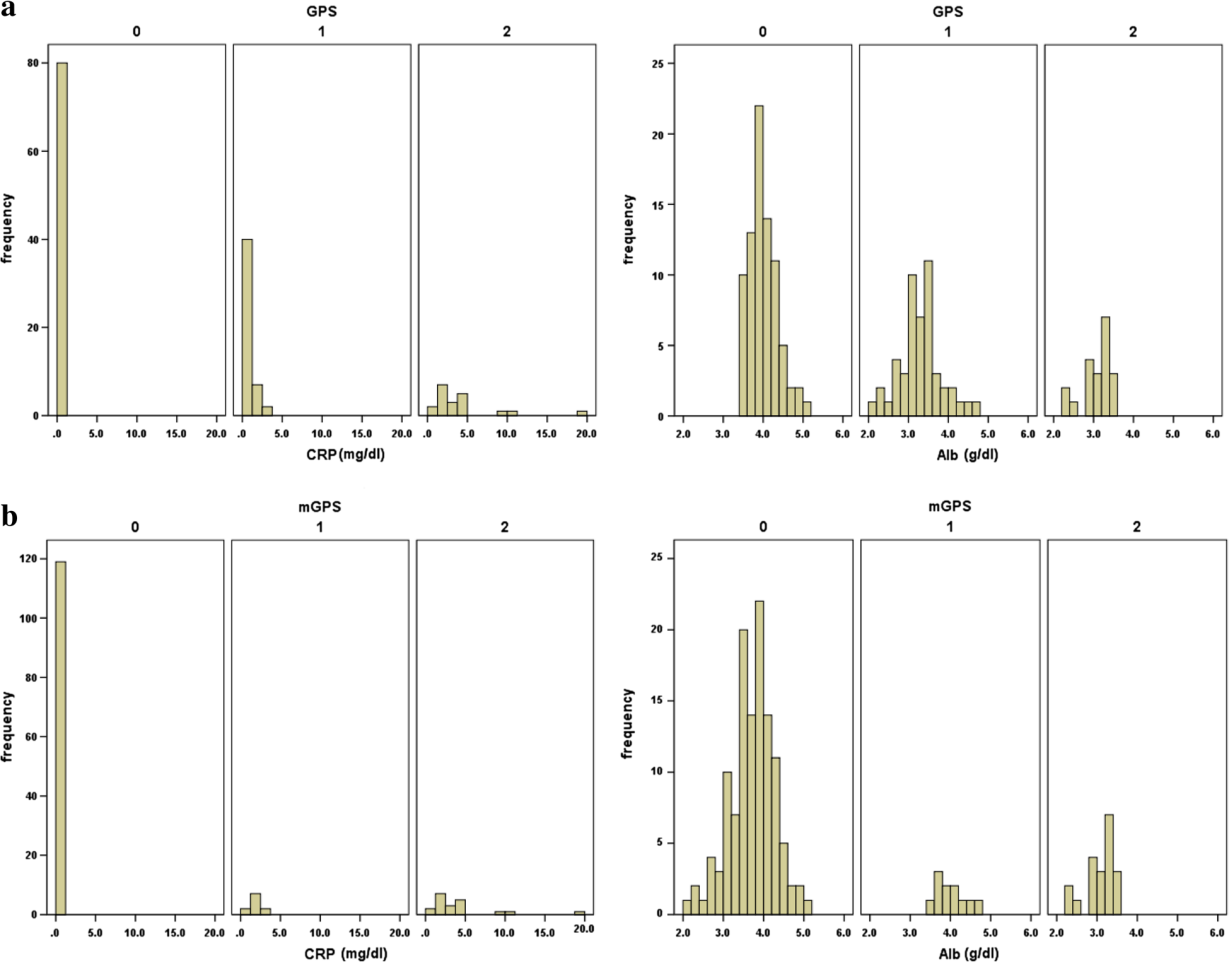
Histogram indicating the distribution of CRP and Albumin in the GPS groups (a), mGPS groups (b).

Thirty one (20.7%) patients showed an elevated CRP level (>1.0 g/dl) and 58 (38.7%) patients had hypoalbuminemia (<3.5 g/dl). Eighty one (54%) patients were allocated a GPS of 0, 49 (32.7%) patients were allocated a GPS of 1, and 20 (13.3%) patients were allocated a GPS of 2, respectively. By contrast, 119 (79.3%) patients were allocated an mGPS of 0, 11 (7.3%) patients were allocated an mGPS of 1, and 20 (13.3%) patients were allocated an mGPS of 2. A histogram indicating the distribution of CRP and albumin in the GPS and m GPS groups is presented in Figure 1a, b.

Baseline characteristics of patients grouped according to GPS/mGPS allocation are shown in Table 1, 2. There were significant differences between the GPS groups in age (P=0.048), AST (P<0.0001), ALT (P=0.017), T-Bil (P<0.0001), PT (P = 0.022), AFP (P=0.008), maximal tumor diameter (P=0.003), tumor number (P=0.041), frequency of vascular invasion (P=0.0002), and frequency of extra hepatic metastasis (P=0.02). An elevated GPS was also associated with higher Child-Pugh scores (P<0.0001), higher CLIP scores (P<0.0001), higher JIS scores (P<0.0001), higher BCLC scores (P<0.0001), and higher TNM classification (P<0.0001).

**Table 1:** Clinicopahtological characteristics of patients grouped according to GPS

**Variable**	**GPS 0 (n = 81)**	**GPS 1 (n = 49)**	**GPS 2 (n = 20)**	**P-value**
Age (years)	71 (43–91)	74 (54–86)	72 (51–79)	0.05
Sex (male/female)	57/23	33/16	16/4	0.57
HBsAg positive (%)	8(10)	4 (8.2)	5 (25)	0.12
HCVAb positive (%)	52 (65)	24 (49)	7 (35)	0.03
AST (IU/l)	50 (18–148)	58 (14–240)	113 (13–384)	<0.0001
ALT (IU/l)	39 (10–202)	39 (12–190)	78 (8–157)	0.02
Total serum bilirubin (mg/dl)	0.7 (0.3-1.9)	1 (0.3-6.1)	1.6 (0.3-8.3)	<0.0001
Albumin (g/dl)	3.9 (3.5-5)	3.2 (2.1-4.6)	3.2 (2.2-3.4)	<0.0001
CRP (mg/dl)	0.1 (0.1-0.8)	0.3 (0.1-2.9)	2.6 (1.1-18.8)	<0.0001
WBC (cells/mm^3^)	5300 (2500–14800)	5100 (1800–14900)	5800 (3900–12400)	0.13
Platelet count (10^4^/mm^3^)	14.2 (2.9-29.5)	11.6 (1.8-42.1)	17.3 (8.7-44.3)	0.07
Prothrombin time (%)	85 (38–100)	78 (41–100)	77 (45–97)	0.02
α-fetoprotein (ng/ml)	17 (1.9-59597)	52 (2–138011)	144 (1.7-280600)	0.01
Child-Pugh grade (A/B/C)	78/2/0	22/25/2	6/10/4	<0.0001
CLIP score (0/1/2/3≧)	35/31/10/4	8/12/17/12	0/7/1/12	<0.0001
JIS score (0/1/2/3≧)	17/32/27/4	0/15/17/17	0/1/6/13	<0.0001
BCLC score (0/A/B/C,D)	16/41/19/4	1/24/14/10	0/6/2/12	<0.0001
Tumor stage (І,ІІ,ІІІ,ІV)	16/34/28/2	4/22/16/7	1/4/4/11	<0.0001
Maximal tumor diameter (mm)	28 (7–90)	37 (10–200)	50 (10–130)	0
Tumor number (solitary/multiple)	45/35	26/23	5/15	0.04
Vascular invasion (absent/present)	77/3	44/5	13/7	0
Extrahepatic metastasis (absent/present)	79/1	47/2	17/3	0.02

Abbreviaions: HBsAg = hepatitis B surface antigen; HCVAb = hepatitis C antibody; AST = aspartate aminotransferase; ALT = alanine aminotransferase.

CRP = C-reactive protein; WBC = white blood cell count; ICG = indocyanine green dye retention rate at 15 minutes; CLIP = Cancer of the Liver Italian Program.

JIS = Japan Integrated Staging score; BCLC = Barcelona Clinic Liver Cancer; GPS = Glasgow Prognostic Score.

**Table 2:** Clinicopahtological characteristics of patients grouped according to mGPS

**Variable**	**mGPS 0 (n = 119)**	**mGPS 1 (n = 11)**	**mGPS 2 (n = 20)**	**P-value**
Age (years)	72 (43–91)	74 (64–82)	72 (51–79)	0.14
Sex (male/female)	80/39	10/1	16/4	0.16
HBsAg positive (%)	12 (10)	2 (18)	5 (25)	0.15
HCVAb positive (%)	76 (64)	1 (9)	7 (35)	0
AST (IU/l)	53 (14–240)	56 (17–238)	113 (13–384)	<0.0001
ALT (IU/l)	39 (10–202)	53 (14–190)	78 (8–157)	0.02
Total serum bilirubin (mg/dl)	0.8 (0.3-3.1)	0.7 (0.3-6.1)	1.6 (0.3-8.3)	0
Albumin (g/dl)	3.7 (2.1-5)	3.9 (3.5-4.6)	3.2 (2.2-3.4)	<0.0001
CRP (mg/dl)	0.1 (0.1-0.9)	1.5 (1.1-2.9)	2.6 (1.1-18.8)	<0.0001
WBC (cells/mm^3^ )	5200 (1800–14900)	5900 (3500–10600)	5800 (3900–12400)	0.07
Platelet count (10^4^/mm^3^ )	13.2 (2.8-30.9)	25.3 (1.8-42.1)	17.3 (8.7-44.3)	0
Prothrombin time (%)	83 (38–100)	95 (74–100)	77 (45–97)	0.01
α-fetoprotein (ng/ml)	22 (1.9-59597)	77 (2–138011)	144 (1.7-280600)	0.05
Child-Pugh grade (A/B/C)	91/26/2	10/1/0	6/10/4	<0.0001
CLIP score (0/1/2/3≧)	41/43/24/11	3/0/3/5	0/7/1/12	<0.0001
JIS score (0/1/2/3≧)	18/43/41/17	0/4/3/4	0/1/6/13	<0.0001
BCLC score (0/A/B/C,D)	18/61/31/9	0/4/2/5	0/6/2/12	<0.0001
Tumor stage (І,ІІ,ІІІ,ІV)	21/52/41/5	0/4/3/4	1/4/4/11	<0.0001
Maximal tumor diameter (mm)	30 (7–200)	110 (40–150)	50 (10–130)	<0.0001
Tumor number (solitary/multiple)	66/53	6/5	5/15	0.04
Vascular invasion (absent/present)	114/5	8/3	13/7	<0.0001
Extrahepatic metastasis (absent/present)	117/2	10/1	17/3	0.01

Abbreviaions: HBsAg = hepatitis B surface antigen; HCVAb = hepatitis C antibody; AST = aspartate aminotransferase; ALT = alanine aminotransferase.

CRP = C-reactive protein; WBC = white blood cell count; ICG = indocyanine green dye retention rate at 15 minutes; CLIP = Cancer of the Liver Italian Program.

JIS = Japan Integrated Staging score; BCLC = Barcelona Clinic Liver Cancer; GPS = Glasgow Prognostic Score.

### Comparison of the prognostic ability for overall survival

The median follow-up duration was 18 (range 1–80) months. At the end of the follow-up period, 77 (51.3%) patients were alive, and 73 (48.7%) patients died. The most common cause of death was tumor progression or hepatic failure (n = 60 , 82.2%), followed by other malignancies (n = 4, 5.5%), gastrointestinal bleeding (n = 3, 4.1%), cardiovascular disease (n = 2, 2.7%), cerebrovascular disease (n = 2, 2.7%), sepsis (n = 1, 1.4%), and pneumonia (n = 1, 1.4%). The 1-year, 3-year, 5-year overall survival rates were 74.1%, 53.3%, and 28.4%, respectively. The 1-year, 3-year, 5-year overall survival rates in the curative treatment group were 95.9%, 71.3%, and 41.4%, respectively. The 1-year, 3-year, 5-year overall survival rates in the non-curative treatment group were 54.9%, 34.7%, and 14.8%, respectively.

**Figure 2:**
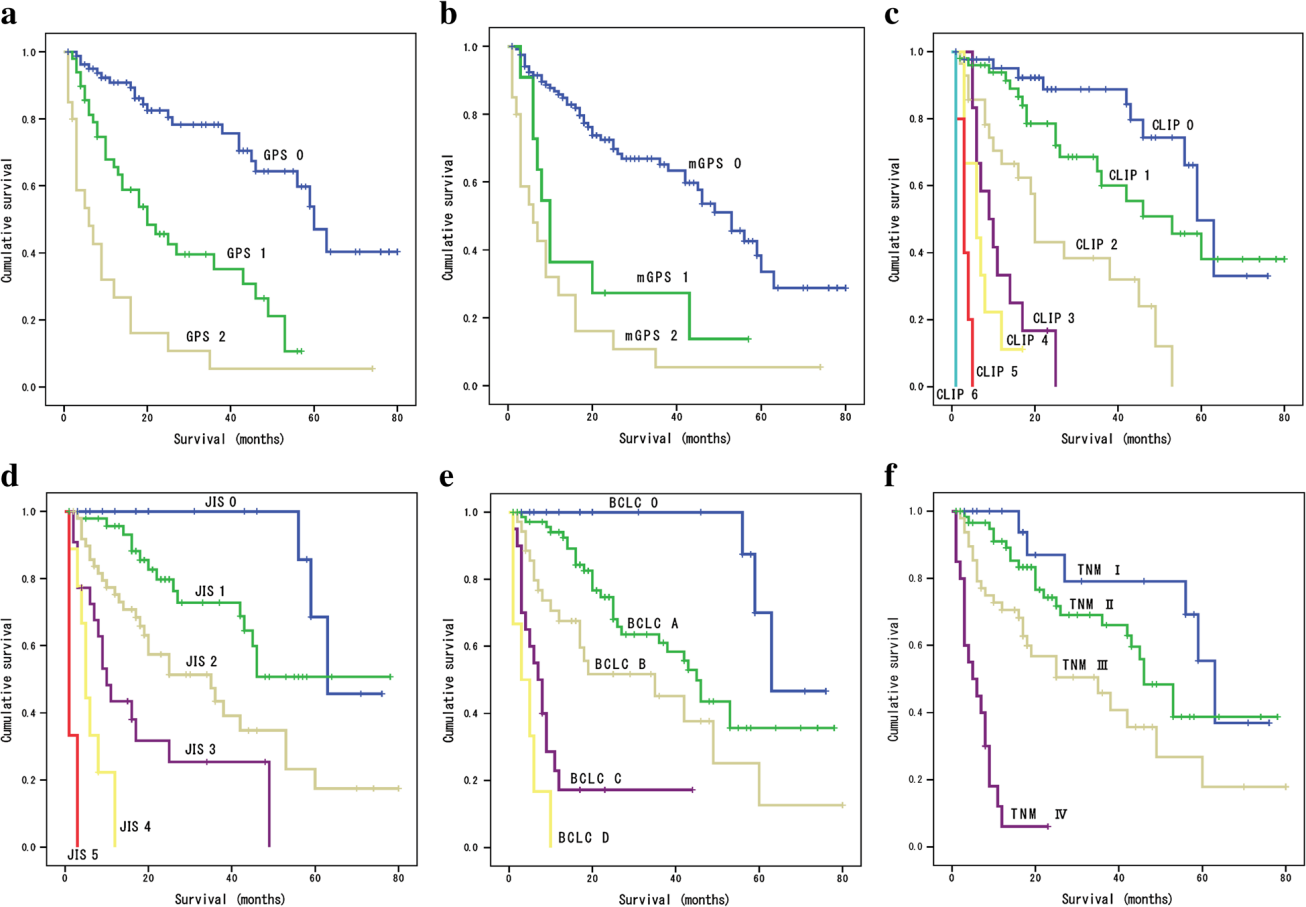
Comparison of cumulative survival according to scoring systems, GPS (a), mGPS (b), CLIP (c), JIS (d), BCLC (e), and TNM (f).

The comparison of overall survival according to the six staging systems is shown in Figure 2a ~ f. A significant difference in overall survival was found across all staging systems (P<0.0001 in all systems). However, no significant difference in survival was found between mGPS 1 versus 2 (P=0.189), CLIP score 0 versus 1 (P=0.133), CLIP score 3 versus 4 (P=0.281), CLIP score 5 versus 6 (P=0.074), JIS score 0 versus 1 (P=0.082), BCLC C versus D (P=0.083), and TNM stage Іversus ІІ(P=0.171). Among the 6 staging systems, only GPS demonstrated significant differences in overall survival between all adjacent strata (P<0.05 in all strata).

Table 3 showed results of the linear trend χ^2^ test and −2 log likelihood for each staging system. In all patients, the CLIP had the highest prognostic power compared to other staging systems in terms of homogeneity and discriminatory ability (linear trend χ^2^ test, 109.31; -2 log likelihood, 545.361). Among patients in the curative treatment group, GPS had the highest prognostic power among all staging systems (linear trend χ^2^ test, 16.33; -2 log likelihood, 169.3).

**Table 3:** Prognostic ability of each staging system

**Patients**	**linear trend χ2 test**	**−2 log likelihood**	**P-value**
**All patients (n = 150)**			
GPS	53.761	588.797	<0.0001
mGPS	46.707	601.175	<0.0001
CLIP	109.309	545.36	<0.0001
JIS	64.461	570.82	<0.0001
BCLC	61.89	577.75	<0.0001
TNM	41.6	589.84	<0.0001
**Curative treatment group (n = 77)**			
GPS	16.33	169.300	<0.0001
mGPS	5.07	177.93	0.03
CLIP	11.28	171.76	0
JIS	9.68	172.22	0
BCLC	N/O	N/O	0.08
TNM	N/O	N/O	0.09
**Non-curative treatment group (n = 73)**			
GPS	23.71	322.27	<0.0001
mGPS	28.815	322.579	<0.0001
CLIP	29.480	311.68	<0.0001
JIS	16.72	326.370	<0.0001
BCLC	20.98	321.9	<0.0001
TNM	16.42	326.7	<0.0001

Abbreviations: GPS = Glasgow Prognostic Score; mGPS = modified Glasgow Prognostic Score;

CLIP = the Cancer of the Liver Italian Program; JIS = the Japan Integrated Staging score;

BCLC = the Barcelona Clinic Liver Cancer; TNM = tumor nodes metastasis classification; N/O = not obtained.

**Figure 3:**
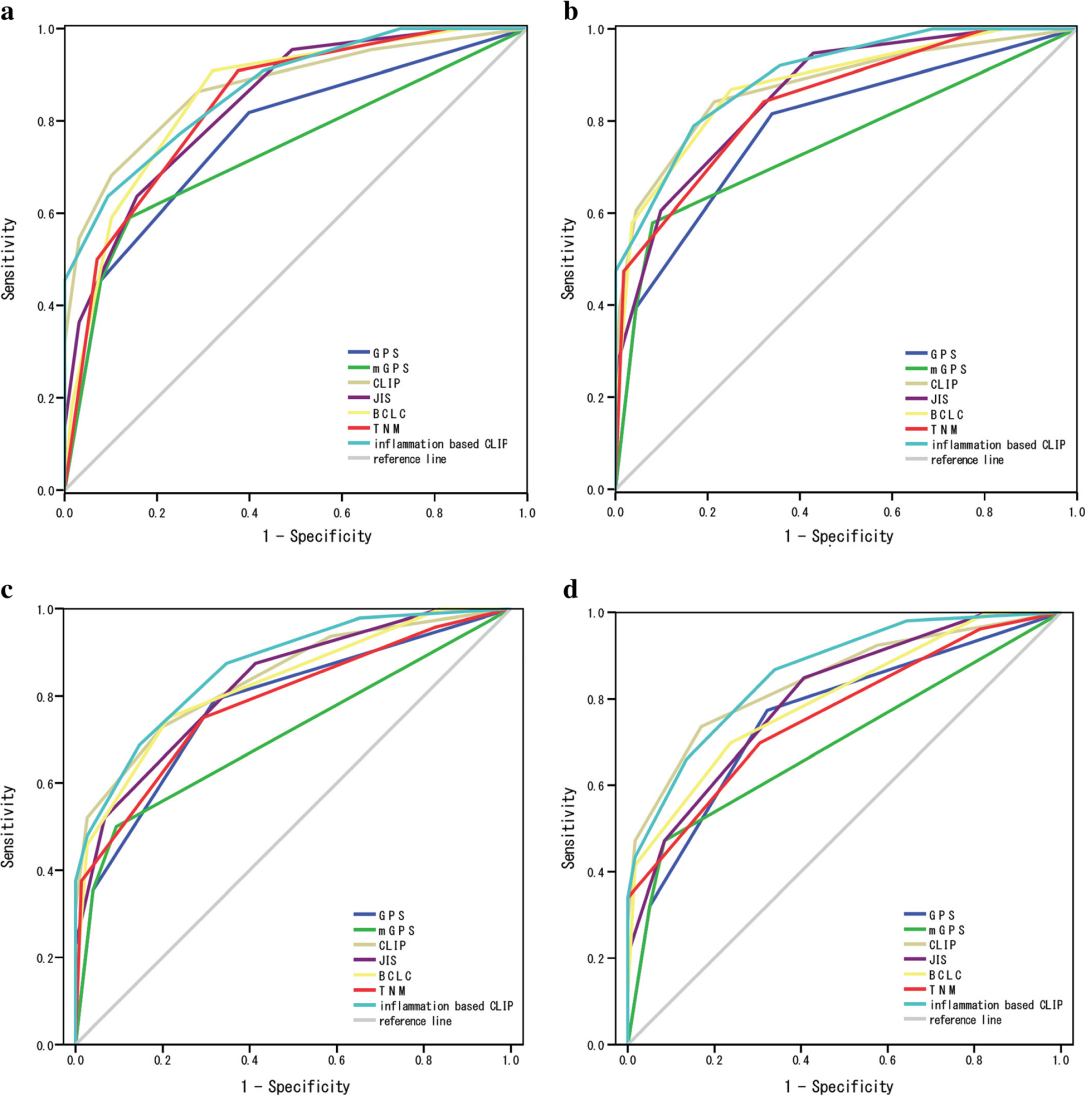
Comparisons of the area under the receiver operating curve for survival status between scoring systems at 6 month (a), 12 month (b), 18 month (c), and 24 month (d).

To assess the discrimination ability of each staging systems, the ROC curves were constructed for survival status at 6-month, 12-month, 18month, and 24-month of follow-up, and the areas under the ROC curve (AUC) were compared (Table 4, Figure 3a ~ d). The CLIP had the highest AUC value among 6 staging systems except for the 12-month value. GPS consistently showed higher AUC values compared with mGPS at each follow-up interval.

**Table 4:** Comparison of the area under the curve between each scoring system at different follow-up intervals

**Period**	**AUC**	**95% CI**	**P-value**
**6-month**			
GPS	0.768	0.655-0.882	<0.0001
mGPS	0.734	0.604-0.864	<0.0001
CLIP	0.871	0.779-0.963	<0.0001
JIS	0.837	0.752-0.922	<0.0001
BCLC	0.85	0.773-0.935	<0.0001
TNM	0.84	0.753-0.921	<0.0001
**12-month**			
GPS	0.79	0.699-0.876	<0.0001
mGPS	0.75	0.650-0.855	<0.0001
CLIP	0.88	0.804-0.948	<0.0001
JIS	0.86	0.794-0.924	<0.0001
BCLC	0.88	0.813-0.946	<0.0001
TNM	0.84	0.771-0.918	<0.0001
**18-month**			
GPS	0.78	0.690-0.864	<0.0001
mGPS	0.710	0.690-0.810	<0.0001
CLIP	0.842	0.768-0.916	<0.0001
JIS	0.83	0.753-0.901	<0.0001
BCLC	0.83	0.753-0.905	<0.0001
TNM	0.79	0.699-0.871	<0.0001
**24-month**			
GPS	0.76	0.667-0.848	<0.0001
mGPS	0.7	0.595-0.795	<0.0001
CLIP	0.84	0.770-0.918	<0.0001
JIS	0.8	0.723-0.882	<0.0001
BCLC	0.800	0.719-0.881	<0.0001
TNM	0.76	0.675-0.852	<0.0001

Abbreviations: AUC = are under the receiver operating curve; 95% CI = 95% confidence interval;

GPS = Glasgow Prognostic Score; mGPS = modified Glasgow Prognostic Score;

CLIP = Cancer of the Liver Italian Program; JIS = Japan Integrated Staging score;

BCLC = Barcelona Clinic Liver Cancer; TNM = tumor nodes metastasis classification.

### Prognostic factors

On univariate analysis, AST (P<0.0001), total serum bilirubin (P<0.0001), albumin (P<0.0001), pretreatment serum CRP level (P<0.0001), Plt (P=0.041), AFP (P<0.0001), GPS (P<0.0001), mGPS (P<0.0001), CLIP score (P<0.0001), JIS score (P<0.0001), BCLC score (P<0.0001), TNM stage (P<0.0001), maximal tumor diameter (P<0.0001), multiple nodules (P<0.0001), vascular invasion (P<0.0001) and extrahepatic metastasis (P=0.001) were associated with the overall survival (Table 5).

**Table 5:** Prognostic factors for overall survival in patients with HCC

	**Univariate analysis**	**Multivariate analysis**	
**Variable**	**P-value**	**Hazard ratio (95% CI )**	**P-value**
Age	0.24		
Sex (male/female)	0.35		
AST	<0.0001		
ALT	0.06		
Total serum bilirubin	<0.0001		
Albumin	<0.0001		
CRP	<0.0001		
WBC	0.42		
Platelet count	0.04		
Prothrombin time	0.09		
α-fetoprotein	<0.0001		
Child-Pugh grade (A/B/C)	<0.0001		
GPS (0/1/2)	<0.0001	1.773 (1.239-2.539)	0.002
mGPS (0/1/2)	<0.0001		
CLIP score (0/1/2/3/4/5/6)	<0.0001	2.243 (1.784-2.820)	<0.0001
JIS score (0/1/2/3/4/5)	<0.0001		
BCLC score (0/A/B/C/D)	<0.0001		
tumour stage (І/ІІ/ІІІ/ІV)	<0.0001		
Maximal tumour diameter (mm)	<0.0001		
tumour number (solitary/multiple)	<0.0001		
Vascular invasion (absent/present)	<0.0001		
Extrahepatic metastasis (absent/prsent)	0		

Univariate and multivariate analysis.

Abbreviaions: AST = aspartate aminotransferase; ALT = alanine aminotransferase; CRP = C-reactive protein;WBC = white blood cell count;

CLIP = Cancer of the Liver Italian Program; JIS = Japan Integrated Staging score; BCLC = Barcelona Clinic Liver Cancer;

GPS = Glasgow Prognostic Score; mGPS = modified GPS.

Due to the correlation between CRP, albumin and GPS and between GPS and mGPS, variables (AST, total serum bilirubin, Plt, AFP, GPS, CLIP score, JIS score, BCLC score, TNM stage, maximal tumor diameter, multiple nodules, vascular invasion and extrahepatic metastasis) were tested in multivariate analysis. Multivariate analysis revealed that only GPS (HR 1.773, 95%CI 1.239-2.539, P=0.002) and CLIP score (HR 2.243, 95%CI1.784-2.820, P<0.0001) were independently associated with overall survival (Table 5). In the curative treatment group, multivariate analysis revealed that GPS (HR 2.068, 95%CI 1.2-3.564, P=0.009) and CLIP score (HR 1.74, 95%CI 1.019-2.971, P= 0.042) were independently associated with overall survival. In the non- curative treatment group, multivariate analysis revealed that total serum bilirubin (HR 1.348, 95%CI 1.02-1.78, P=0.036) and CLIP score (HR 1.962, 95%CI 1.456-2.645, P <0.0001) were independently associated with overall survival.

When GPS was combined to the CLIP score to form a new prognostic system, named by the authors as inflammation-based CLIP, the new system provided better prognostic accuracy with linear trend χ^2^ test 115.129, -2 log likelihood 539.132, and the area under the ROC 0.869 at 6-month, 0.897 at 12-month, 0.865 at 18month, and 0.862 at 24-month.

## Discussion

In the present study, we have demonstrated that GPS, an inflammation-based prognostic score, is associated with tumor progression and reduced liver function and can be considered an independent marker of poor prognosis in patients with HCC notwithstanding the disease stage and/or liver functional status along with the CLIP score. Moreover, GPS proved to be more suitable than mGPS in patients with HCC with regard to its discriminating ability and the monotonicity of gradients.

Links between cancer and inflammation were first established in the nineteenth century and that were based on observations that tumors often arose at sites of chronic inflammation and that inflammatory cells are present in biopsied samples collected from tumor tissues. Today, it is well known that cancer promotes release of proinflammatory cytokines from tumor cells. The cytokines interact with immunovascular system and facilitate cancer growth, invasion, and metastasis [23,24].

Recent studies have shown that elevated serum CRP levels may be associated with tumor size, distant metastasis, vascular invasion, lymph node metastasis and tumor recurrence, resulting in poor prognosis in patients with various cancers, including HCC [16,25].

It has been also reported that serum albumin participate in systemic inflammatory response and that decline of its serum level is a poor prognostic factor for long-term survival in patients with various cancers [8,19].

Based on these reports, GPS, incorporating CRP and serum albumin levels, may reflect both presence of the systemic inflammatory response (CRP), and the progressive nutritional decline (albumin) in patients with cancers, resulting in poor survival outcome [20].

Consistent with the Ishizuka’s report [14], our study demonstrates that an elevated GPS is associated with factors of tumor progression such as: maximal tumor diameter, tumor number, vascular invasion, extra hepatic metastasis, higher CLIP scores, higher JIS scores, higher BCLC scores and higher TNM classification grade. In addition, our data show that an elevated GPS is also associated with factors indicating reduced liver function such as higher T-Bil, lower albumin, higher ICG and higher Child-Pugh scores. This is partly because, unlike in the Ishizuka’s report, in our study we enrolled patients not eligible for surgical resection: with more advanced stages of the disease and reduced liver function.

Although significant differences in overall survival were found across all staging systems, such differences were not observed between mGPS 1 versus 2, CLIP score 0 versus 1, CLIP score 3 versus 4, CLIP score 5 versus 6, JIS score 0 versus 1, BCLC C versus D, and TNM grade Іversus ІІ, suggesting poorer discrimination ability in early and advanced stages of the disease. Only GPS demonstrated significant differences in overall survival between all adjacent strata, indicating that GPS could discriminate between early and advanced stages of the disease.

Linear trend χ^2^ test and −2 log likelihood calculated using the Cox model showed that GPS has fairly good ability of discriminating the survival of patients in different HCC stages and has greater homogeneity of survival among patients within the same stage, particularly in the curative treatment group, suggesting that it can provide better prognostic power than other scoring systems in the setting of curative treatment.

On multivariate analysis, GPS was independently associated with overall survival along with the CLIP score, which is consistent with the Ishizuka’s report [14]. However, our results indicate that GPS is an independent marker of poor prognosis in patients with HCC in various stages of disease and different liver functional statuses. By contrast, JIS, BCLC, and TNM were not found to be independent poor prognostic factors. A scoring system should be simple and easy to apply for prognosis prediction before treatment is initiated. In this regard, GPS can be a useful tool for prognostication and stratification of patients with HCC, because being based on only 2 laboratory data, CRP and albumin, it is conventionally available without additional imaging techniques or histological examinations before commencing treatment [7].

The Cox model and AUC analysis showed GPS is more suitable than mGPS for patients with HCC with regard to discriminating ability and the monotonicity of gradients. Recently, in a Glasgow Inflammation Outcome Study, Proctor et al. have shown that mGPS was a powerful prognostic factor independent of tumor site in patients with cancer and was superior to GPS in the greater consistency and more general use [12]. Their observations were based on the results that a low albumin concentration alone was uncommon (<10% of all patients) and was not significantly associated with cancer-specific survival in many cancers including hepatopancreaticobiliary cancer (P = 0.209). However, our study included 38 (25.3%) patients with low albumin concentration alone. Furthermore, serum albumin is one of the components of the Child-Pugh Classification and hypoalbuminemia has been reported as an independent poor prognostic factor in patients with HCC [26]. Moreover, in the Glasgow Inflammation Outcome Study, hepato-pancreatico-biliary cancer included pancreatic and biliary tract cancers besides HCC. Therefore, we speculate that GPS is more suitable than mGPS for patients with HCC.

A number of scoring systems for HCC have been proposed to date. However, controversy remains about which system is best at predicting survival of HCC patients. Characteristics of tumor-related variables, the relative score weighted for each variable, preferred treatment modality in different centers, the numbers of analyzed patients, and the etiology of liver diseases could contribute to this controversy [27]. In the current study, the CLIP system proved to be the best prognostic model for HCC with regard to discrimination ability and the monotonicity of gradients. This result is consistent with a previous study with HCC patients undergoing palliative TACE and another study with advanced HCC patients [2,26]. The CLIP system was originally derived from unselected patient population and the majority of them had received non-surgical treatment [4]. Therefore, it is generally accepted that the CLIP system may be more suitable for predicting the survival of HCC patients who receive non-surgical treatments than BCLC system or JIS system [26,28]. In our study, many patients (n = 141, 94%) were treated non-surgically, which may be an important reason why the CLIP was superior to the BCLC or the JIS in predicting survival. However, a recent study from Taiwan has demonstrated that the CLIP system was the best prognostic model for HCC in terms of prognostic stratification for patients from early to advanced cancer stage irrespectively with curative or non-curative treatments [27]. This result indicates that prediction accuracy of the CLIP system is highly stable and is independent of the treatment strategy. When GPS was combined to the CLIP system to form a new prognostic system, named inflammation-based CLIP provided even better prognostic accuracy than GPS alone, suggesting that addition of GPS could improve the discriminatory ability of the CLIP system.

The current study has some limitations. First, it was a retrospective, small sample size, single-center study. Second, the majority of patients enrolled were treated non-surgically. Our results may not be applicable to surgically oriented centers or high volume centers with frequently performed liver transplantations. Third, the therapeutic effects of each treatment method were not included into prognostic factors’ evaluation. Since many patients received multiple treatment sessions due to tumor recurrence during their follow-up periods (tumor progression and worsening liver functional reserve), it was difficult to evaluate all therapeutic effects as prognostic factors in this patient population. Fourth, there may be the potential causal relationships between liver function and inflammation. As progressive HCC patients would have poor liver function and reduced albumin, GPS in this group of patient is no longer only a marker of inflammation but also a marker of poorer liver function. However, Cervoni et al. have demonstrated that a systemic inflammation response, as evidenced by an elevated CRP concentration, is associated with poor survival in Child Pugh score > B8 cirrhotic patients independently of Model of End Stage Liver Disease [29]. We also have shown that CRP is an independent marker of poor prognosis in patients with HCC, irrespective of tumor stage and liver function [16].

## Conclusion

Our study demonstrated that GPS is associated with tumor progression and reduced liver function and can be considered as an independent marker of poor prognosis in patients with HCC in various stages of disease and different liver functional status along with the CLIP score. GPS is more suitable than mGPS for patients with HCC with regard to discriminating ability and the monotonicity of gradients.

## Competing interests

The authors declare that they have no competing interests.

## Authors’ contributions

AK, HO, and NF participated in the design of the study. NI, AI, MO, KT, and CS carried out acquisition of data. AK has been involved in drafting the manuscript. KK and MM performed the statistical analysis. HN and HT have been involved in revising it critically for important intellectual content. All authors read and approved the final manuscript.

## Pre-publication history

The pre-publication history for this paper can be accessed here:

http://www.biomedcentral.com/1471-2407/13/52/prepub
